# Novel Insight into the Role of Endoplasmic Reticulum Stress in the Pathogenesis of Myocardial Ischemia-Reperfusion Injury

**DOI:** 10.1155/2021/5529810

**Published:** 2021-03-26

**Authors:** Hang Zhu, Hao Zhou

**Affiliations:** ^1^Institute of Geriatric Cardiovascular Disease, Medical School of Chinese People's Liberation Army, China; ^2^Center for Cardiovascular Research and Alternative Medicine, University of Wyoming College of Health Sciences, USA

## Abstract

Impaired function of the endoplasmic reticulum (ER) is followed by evolutionarily conserved cell stress responses, which are employed by cells, including cardiomyocytes, to maintain and/or restore ER homeostasis. ER stress activates the unfolded protein response (UPR) to degrade and remove abnormal proteins from the ER lumen. Although the UPR is an intracellular defense mechanism to sustain cardiomyocyte viability and heart function, excessive activation initiates ER-dependent cardiomyocyte apoptosis. Myocardial ischemia/reperfusion (I/R) injury is a pathological process occurring during or after revascularization of ischemic myocardium. Several molecular mechanisms contribute to the pathogenesis of cardiac I/R injury. Due to the dual protective/degradative effects of ER stress on cardiomyocyte viability and function, it is of interest to understand the basic concepts, regulatory signals, and molecular processes involved in ER stress following myocardial I/R injury. In this review, therefore, we present recent findings related to the novel components of ER stress activation. The complex effects of ER stress and whether they mitigate or exacerbate myocardial I/R injury are summarized to serve as the basis for research into potential therapies for cardioprotection through control of ER homeostasis.

## 1. Introduction

Myocardial ischemia/reperfusion (I/R) injury occurs when myocardial tissues or cardiomyocytes are resupplied with fresh blood flow following a period of ischemia. In that situation, tissues/cells not only fail to recover from the ischemic damage but also develop additional injury caused by the reperfusion itself [1, 2]. This phenomenon is particularly prominent in the heart, liver, and brain [3–5]. Clinically, cardiac surgery and coronary artery bypass graft may cause myocardial I/R injury [6–8]. It is now generally believed that the main mechanisms of reperfusion injury are excessive formation of free radicals within the tissue and intracellular calcium overload [9–11]. Among the various biochemical mechanisms and signal pathways that may be involved [12–14], endoplasmic reticulum (ER) stress has been found to be associated with reperfusion-mediated oxidative stress and cardiomyocyte death [15, 16]. ER stress refers to a pathological process associated with hypoxia, starvation, calcium imbalance, and free radical overproduction that disrupts the physiological functions of the ER [17, 18]. These stimuli may cause signaling from the ER to the cytoplasm and nucleus, where adaptive responses or the apoptotic program will be ultimately activated [19, 20].

Recent studies have reported a close relationship between ER stress and cardiac I/R injury [21, 22]. This suggests reducing ER stress through genetic approaches or pharmacological treatments could potentially reduce myocardial I/R injury [23–25], thereby bringing clinical benefits on many patients with cardiovascular disease. This review focuses on the current research investigating the role played by ER stress in myocardial I/R injury with the aim of identifying clinical approaches that may be applied to reduce cardiac I/R injury in the future.

## 2. Overview of Myocardial I/R Injury

Nutrients are supplied to tissues and metabolic waste carried away by the circulation. Insufficient blood flow to a tissue, such as the myocardium, results in ischemia [26, 27], which can lead to cell death and tissue damage. Myocardial ischemia is usually caused by occlusion of one or more coronary arteries, which is followed by a decline in oxygen tension within the myocardium [2, 28, 29]. Myocardial ischemia severely hinders oxidative metabolism of fatty acids, glucose, pyruvate, and lactic acid, which causes energetic stress within cardiomyocytes [30, 31]. It also slows or even stops mitochondrial respiration [32, 33], diminishing oxidative phosphorylation and ATP production. In the absence of sufficient oxygen, ATP production through glycolysis is enhanced, which leads to overproduction of lactic acid [34, 35] and, in turn, intracellular acidosis. In addition, ischemia interrupts *β*-oxidation of fatty acids and thus promotes accumulation of incomplete fatty acid metabolites in the cytoplasm [36, 37]. The most important change within the ischemic myocardium is the reduced generation of high-energy phosphoric compounds (e.g., ATP) and cardiomyocyte death due to ATP deficiency [38, 39]. As a result of the ATP undersupply, the calcium pump within cardiomyocytes cannot effectively remove calcium from the cytoplasm, resulting into calcium overload [40, 41]. The resultant abnormal calcium signal blunts ventricular contraction and promotes the development of cardiac dysfunction [42–44].

From the perspective of treatment, timely restoration of blood flow to the myocardium is an effective way to relieve tissue ischemia and insufficient nutrient supply [45, 46]. Interestingly, however, reperfusion of ischemic tissue can cause additional damage due to I/R injury [47, 48]. This concept was first proposed in 1955 by Sewell et al., based on observations made in dogs after coronary artery ligation [49]. They reported that removing the coronary ligation, and thus restoring of myocardial perfusion, induced ventricular fibrillation and death [50, 51]. This concept was further validated in 1960 by Jennings et al. [52], who reported that when tissue or cells regain a blood supply after transient ischemia, they undergo I/R injury. It was also shown that myocardial ischemia and subsequent reperfusion injury are independent but interrelated pathophysiological processes [53, 54]. Consequently, the prevention and treatment of reperfusion injury should start during the ischemic period, and the ischemia must be removed as soon as possible [55, 56]. The shorter the duration of ischemia, the smaller are the ischemic changes and the possibility of injury after reperfusion [57, 58]. At present, there is no particularly effective way to cope with myocardial I/R injury [59–61]. Several studies have been conducted to understand the molecular mechanisms underlying myocardial I/R injury. Oxidative stress, microvascular damage, inflammatory responses, autophagy inhibition, immune disorders, platelet activation, cardiomyocyte metabolic disturbance, ER stress, and mitochondrial dysfunction are all reported to be potential pathological factors contributing to the development of cardiac I/R injury [62–66].

## 3. Molecular Basis of ER Stress

### 3.1. Overview of the ER

The ER is a membranous tubular organelle within eukaryotic cells [67]. It is found in two forms: rough and smooth [68]. Rough ER localizes with ribosomes and is mainly responsible for protein folding and posttranslational modification [69, 70]. Smooth ER, on the other hand, functions to maintain lipid biosynthesis and calcium storage [71]. ER stress is a state in which an external stimulus disrupts ER homeostasis and triggers the accumulation of unfolded or misfolded proteins within the ER lumen [72]. Calcium overload and abnormal lipid metabolism, due to ER dysregulation, will further promote ER stress [73]. The stimuli thought to cause ER stress include nutritional deficiency, hypoxia, ischemia, oxidative stress, and DNA damage [74–76]. When ER stress occurs, the cell reduces protein synthesis and promotes degradation of misfolded proteins [77]. However, under continuous strong stimulation, excessive ER stress is associated with cell apoptosis [78].

### 3.2. Activation of ER Stress

**Figure 1:**
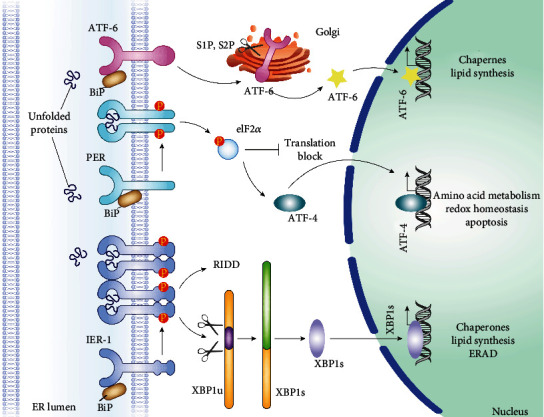
The regulatory mechanisms of endoplasmic reticulum (ER) stress. ER stress in mammals has four components: inhibition of protein translation, upregulation of molecular chaperones, activation of the protein degradative program, and induction of apoptosis [79]. ER stress signal transduction is mediated via three crucial enzymes [80]: protein kinase R-link ER kinase (PERL), activating transcription factor-6 (ATF-6), and inositol-requiring enzyme-1 (IRE1). ER molecular chaperones acting as sensors of ER homeostasis play a key role in monitoring the accumulation of unfolded proteins within the ER.

ER stress in mammals has four components: inhibition of protein translation, upregulation of molecular chaperones, activation of the protein degradative program, and induction of apoptosis [79]. ER stress signal transduction is mediated via three crucial enzymes (Figure 1) [80]: protein kinase R-link ER kinase (PERL), activating transcription factor-6 (ATF-6), and inositol-requiring enzyme-1 (IRE1). ER molecular chaperones acting as sensors of ER homeostasis play a key role in monitoring the accumulation of unfolded proteins within the ER [81]. Under physiological conditions, GRP78 (also known as binding immunoglobulin protein; BiP) binds to PERK, ATF-6, and IRE1 [82] within the ER. However, GRP78 has greater affinity for unfolded proteins; consequently, when ER homeostasis is disrupted, leading to accumulation of unfolded proteins within the ER, GRP78 dissociates from PERK, ATF-6, and IRE1, which results in the activation of ER stress signaling transduction pathways [83].

### 3.3. The Transduction Pathways of ER Stress

#### 3.3.1. PERK Pathway

PERK is a transmembrane protein in the ER membrane [84]. After dissociation of GRP78, it forms a homodimer and is then activated by autophosphorylation. Phosphorylated PERK catalyzes the phosphorylation of eukaryotic initiation factor-2*α* (elF2*α*) [85], which inactivates eIF2*α*-mediated translation. This effect significantly represses the transcription of most mRNA and, in turn, protein synthesis, which reduces the protein load on the ER [86]. Interestingly, elF2*α* phosphorylation is associated with an increase in the transcription of activating transcription factor-4 (ATF-4), which, after translation, translocates the cell nucleus and functions to upregulate ER molecular chaperones [87]. However, if ER homeostasis cannot be restored, the continuous overexpression of ATF-4 will promote the upregulation of C/EBP homologous protein (CHOP), a potential proapoptotic protein regulating cell death [88].

#### 3.3.2. ATF-6 Pathway

Like PERK, ATF-6 is an ER transmembrane protein [89]. After dissociation of GRP78, ATF-6 translocates to the Golgi apparatus where it is cleaved and activated by the proteases Sit-1/2. The activated ATF-6 migrates into the nucleus where it forms homodimers or heterodimers with other transcription factors, leading to the upregulation of ER chaperone genes [90].

#### 3.3.3. IRE1 Pathway

Upon dissociation of GRP78, the ER transmembrane protein IRE1 forms a homodimer and undergoes autophosphorylation activation [91]. Activated IRE1 has endoribonuclease activity [92], which can cut the mRNA encoding XBP1 (x-box binding protein-1) to form a new transcript encoding a second XBP1 isoform [93]. When abundant, the translated XPB1 protein migrates into the nucleus, where it upregulates the expression of genes related to ER stress [94]. Long-term activation of the IRE1 is associated with apoptosis activated via the TRAF2/ASK1/JNK pathway.

### 3.4. Unfolded Protein Response

After synthesis on the ribosomes, proteins must be folded and packaged correctly within the ER. Protein folding is carried out under redox conditions and requires two ER stress reactive proteins [95]: ER stress oxidoreductase (ERO) and disulfide proteolytic enzyme. After dissociation of GRP78 and their autoactivation (as described above), IRE1, ATF-6, and PERK respond to the presence of incorrectly folded proteins associated with ER stress [96, 97]. This is called the “unfolded protein response” (UPR) [98]. A key function of the ER is identification, control, and correction of protein quality. Proteins that cannot be folded correctly will be transferred from ER to the cytoplasm for degradation by the 26S proteasome [99]. The early stage of the URP is the activation of proteasome-induced degradation of unfolded proteins and the upregulation of XBP1 and ATF-4 [100]. These alterations are aimed at reducing the load of unfolded or misfolded proteins within ER. Later, an inflammatory response is activated via NF-*κ*B and JNK [101], which enhances defensive responses within the cytoplasm. If these responses are unable to restore ER function or cell homeostasis, the cell apoptosis program will be activated as the final stage of the UPR.

## 4. Role of ER Stress in Myocardial I/R Injury

### 4.1. ER Stress and Calcium Overload

Myocardial contraction relies on the oscillation of cytoplasmic free calcium concentration. Within cardiomyocytes, smooth ER (termed sarcoplasmic reticulum; SR) contains the primary calcium store. Excessive calcium release from SR into the cytoplasm leads to intracellular calcium overload, which is closely associated with cardiomyocyte contraction dysfunction and cell death [102–104]. ER dysfunction-mediated calcium overload plays an important role in myocardial I/R injury. During reperfusion, the function of the sodium-calcium exchanger and L-type calcium channels is impaired as a result of the insufficient oxygen supply during the ischemia [105]. By contrast, the expression of calcium-sensitive receptors, such as 1,4,5-inositol trisphosphate receptor (IP3R), is significantly increased due to hypoxic stress or upregulation of hypoxia-inducible factor-1 (HIF1) [106]. These effects enhance calcium release from SR into the cytoplasm under conditions where physiological extrusion of calcium from the cell is suppressed. Thus, ER stress is an upstream trigger of cardiomyocyte calcium overload.

### 4.2. ER Stress and Cardiomyocyte Apoptosis

**Figure 2:**
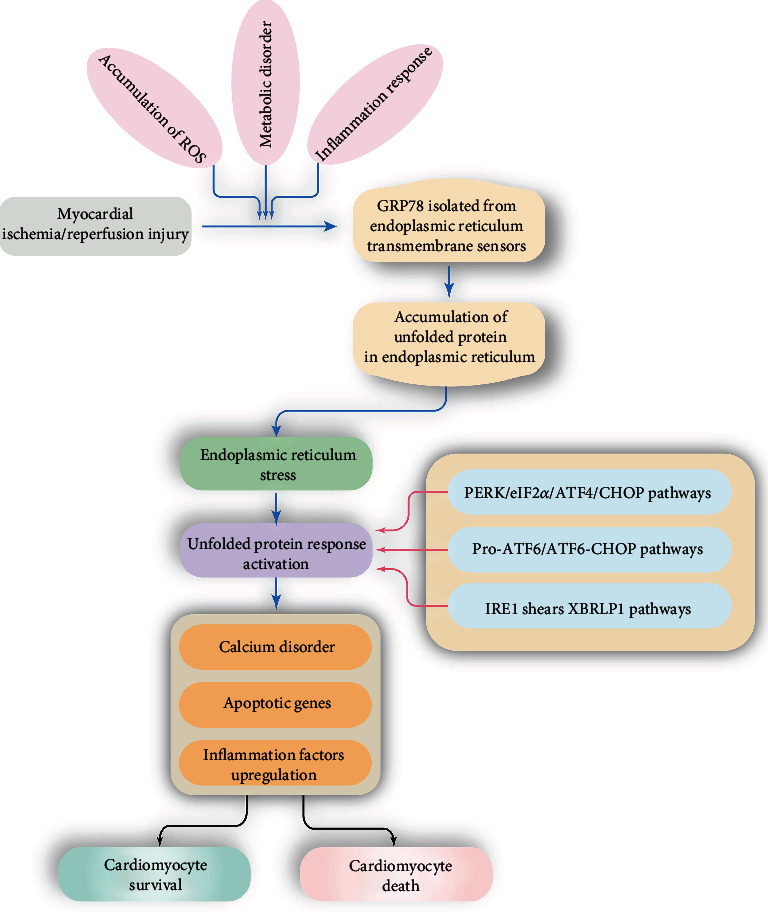
Role of endoplasmic reticulum (ER) stress in myocardial ischemia/reperfusion injury. ER stress is activated by accumulation of ROS, metabolic disorder, or inflammation response, which is featured by GRP78 isolation from ER. Then, unfolded protein accumulation in ER will activate the unfolded protein response (UPR) which is followed by calcium disorder, apoptotic gene upregulation, and inflammation response, resulting into cardiomyocyte death or survival dependent on the extent of ER stress.

Once UPR fails to control the level of unfolded or misfolded proteins, ER stress will trigger the activation of apoptotic signaling. It is currently believed that ER stress can cause cardiomyocyte apoptosis via three pathways (Figure 2).

#### 4.2.1. CHOP Pathway

CHOP is a transcription factor belonging to the C/EBP family. Under normal circumstances, CHOP expression is very low. The transcription and translation of CHOP are primarily regulated by IRE1*α*, ATF-6, and PERK [107, 108], and CHOP plays a key role in ER-induced apoptosis, such as that induced by I/R injury [109]. Upregulation of CHOP induces the expression of a variety of downstream proapoptotic and antiapoptotic genes, including Bcl-2, Bax, Bim, growth arrest and DNA damage-inducible protein 34 (GADD34), ER oxidoreductase-1*α* (ERO1*α*), and the death receptor 5 (DR5) [110]. Among those, GADD34 promotes the expression of protein phosphatase-1 (PP1), which in turn augments transcription of genes related to UPR [111]; ERO1*α* triggers calcium leakage from the ER through IP3Rs, which leads to calcium overload-dependent cell apoptosis [112, 113]; and DR5 triggers apoptosis through activation of caspase-8 [114].

#### 4.2.2. IRE1*α*/JNK Pathway

IRE1*α* is a component of the most conserved pathway in mammalian UPR [115]. It has two active enzyme domains: a serine/threonine kinase domain and an endoribose nuclease (RNase) domain. When ER stress is induced, unfolded or misfolded proteins in the ER lumen directly bind to and activate IRE1*α*. Once activated, IRE1*α* recruits tumor necrosis factor receptor-related factor-2 (TRAF2) and apoptotic-signaling kinase-1 (ASK1) [116], after which JNK is phosphorylated by the resultant IRE1*α*-TRAF2-ASK1 signaling complex [117, 118]. Following cardiac I/R injury, activated JNK may promote cardiomyocyte apoptosis through phosphorylation of various members of the Bcl-2 family [119, 120]. For example, JNK catalyzes phosphorylation of the antiapoptotic protein Bcl-2, which impairs its activity. At the same time JNK catalyzed, phosphorylation enhances the proapoptotic activity of Bim [121]. These alterations work together to mediate apoptosis in cardiomyocytes.

#### 4.2.3. The Caspase-12 Pathway

The caspase-12 pathway is considered to be an ER-specific, nonmitochondrial-dependent apoptotic pathway [122]. Caspase-12 activation is also a feature of ER stress-mediated cardiomyocyte apoptosis [123]. Under normal circumstances, caspase-12 binds to the ER membrane and forms a complex with TRAF2. ER stress directly induces caspase-12 dissociation from the ER membrane, enabling it to be activated by calpain [124, 125] or the IRE1*α*-TRAF2 complex [126]. Once activated, caspase-12 cleaves and activates caspase-9, which in turn cleaves and activates caspase-3 to promote apoptosis [127].

## 5. Summary and Outlook

ER stress arises via multiple signaling pathways, expression of multiple genes, and participation of multiple stress factors. In cases of mild or early myocardial injury, ER stress involves a variety of protective proteins, which reduce the pathological stress on cardiomyocytes. However, excessive ER stress is associated with protein quality control disorder, resulting in the upregulation of apoptotic proteins. Notably, the role of ER stress during ischemia differs from that during reperfusion. It remains unclear whether ER stress is protective in the ischemic heart and only becomes lethal following reperfusion. In addition, although the molecular mechanisms underlying ER stress and its role in I/R injury have been characterized, the interactive effects of ER stress and other pathological alterations that occur during cardiac I/R injury, such as oxidative stress and mitochondrial dysfunction, are still not fully understood. Moreover, there are still no specific drugs targeting ER stress available in clinical practice. Additional investigations are therefore required to help us better understand the role of ER stress in myocardial I/R injury.

## Data Availability

All data generated or analyzed during this study are included in this published article.
